# Sex-specific features of spine densities in the hippocampus

**DOI:** 10.1038/s41598-020-68371-x

**Published:** 2020-07-09

**Authors:** Nicola Brandt, Tobias Löffler, Lars Fester, Gabriele M. Rune

**Affiliations:** 1 Institute of Neuroanatomy, Center for Experimental Medicine, University Medical Center Hamburg-Eppendorf, Martinistr. 52, 20246 Hamburg, Germany; 2 Present Address: Department of Human Medicine, Division of Anatomy, School of Medicine and Health Sciences, Carl Von Ossietzky University Oldenburg, Carl-von-Ossietzky Str. 9-11, 26129 Oldenburg, Germany; 3 Present Address: Institute of Anatomy and Cell Biology, Friedrich-Alexander-Universität (FAU) Erlangen-Nürnberg, Krankenhausstr. 9, 91054 Erlangen, Germany

**Keywords:** Cellular neuroscience, Synaptic development

## Abstract

Previously, we found that in dissociated hippocampal cultures the proportion of large spines (head diameter ≥ 0.6 μm) was larger in cultures from female than from male animals. In order to rule out that this result is an in vitro phenomenon, we analyzed the density of large spines in fixed hippocampal vibratome sections of Thy1-GFP mice, in which GFP is expressed only in subpopulations of neurons. We compared spine numbers of the four estrus cycle stages in females with those of male mice. Remarkably, total spine numbers did not vary during the estrus cycle, while estrus cyclicity was evident regarding the number of large spines and was highest during diestrus, when estradiol levels start to rise. The average total spine number in females was identical with the spine number in male animals. The density of large spines, however, was significantly lower in male than in female animals in each stage of the estrus cycle. Interestingly, the number of spine apparatuses, a typical feature of large spines, did not differ between the sexes. Accordingly, NMDA-R1 and NMDA-R2A/B expression were lower in the hippocampus and in postsynaptic density fractions of adult male animals than in those of female animals. This difference could already be observed at birth for NMDA-R1, but not for NMDA-R2A/B expression. In dissociated embryonic hippocampal cultures, no difference was seen after 21 days in culture, while the difference was evident in postnatal cultures. Our data indicate that hippocampal neurons are differentiated in a sex-dependent manner, this differentiation being likely to develop during the perinatal period.

## Introduction

Synapse formation and elimination are critical in the establishment of neuronal circuitries and normal brain function. Dendritic spines are the postsynaptic partners of most excitatory synapses in the CNS, and are commonly taken to be indicative of synapse formation. The critical role of spines in the formation and storage of memories is widely established, and consistent changes in spine number and morphology have been found in a number of neurological and psychiatric disorders^1^ and during normal aging^2^.

Spines are motile^3^, highly dynamic and can be formed and disappear within very short periods of time^4^, thus underlining their prominent role in synaptic plasticity. Spine dynamics are reflected by various spine types, which are discernible at any point in time: these types are filopodia, which are long cytoplasmic protrusions, believed to represent immature spines; stubby spines, which lack a spine head; thin spines with a neck and a small head, and mushroom spines, with a large spine head originating from a spine neck, which are considered to represent mature spines^5,6^. Since large spines, defined by a head diameter of ≥ 0.6 μm, have larger postsynaptic densities that anchor more glutamate receptors than thin spines, some authors classify them as “memory” spines, while thin spines are categorized as “learning” spines^7–9^. As yet, specific functions of other spines, such as filopodia and stubby spines have been discussed less in relation to specific functions.

80% of the large spines contain a spine apparatus, which has been considered a spine-specific Ca^2**+**^ store^10–14^. Synaptopodin, a protein linked to calcium stores^15^, is closely associated with the spine apparatus^16,17^ and is required for its formation. In synaptopodin knock-out mice, which lack a spine apparatus, LTP is impaired and the mutants show behavioural deficits. Since large spines commonly contain a spine apparatus, the data underscore the particular role of large spines in memory.

Spine and spine synapse density in the hippocampus depend on sex steroid hormones, such as estradiol and testosterone, originating from the gonads^18,19^, and/or from local synthesis in the hippocampus^20–22^. Increasing evidence, however, suggests that estrogen-induced spine formation relies on local synthesis in the brain (for review see^22^). In the female rat hippocampus spine density varies with the estrus cycle^18,21^, which is presumably mediated by the Gonadotropin Releasing Hormone, which regulates estradiol synthesis in the hippocampus^21,23^. Hence, cyclicity in female animals hinders the comparison of spine density between males and females. Sex differences in performance of various cognitive tasks, however, which should be mirrored by structural differences, have been frequently described^24–28^.

We recently found that in dissociated hippocampal cultures the proportion of large spines, thus spines with a head diameter ≥ 0.6 μm, was larger in cultures originating from female animals than in cultures generated from male animals^29^. This finding prompted us to test whether this difference also exists in vibratome sections of animals that had been perfused for fixation immediately before removal of the hippocampus. For our experiments, we used Thy1-GFP transgenic mice^30^, in which GFP is expressed only in subpopulations of neurons and thus allows the counting of spines in hippocampal vibratome sections on the light microscopical level. Data on spine synapse density in EM micrographs in male and female animals and in hippocampal slice cultures, as well as in dissociated hippocampal cultures of male and female animals, has been previously published^18–20,26,31^. These studies revealed that male and female animals respond differently to sex neurosteroids, i.e. sex steroids synthesized in the brain. Estradiol was shown to be essential for the maintenance of synaptic density and long-term potentiation (LTP) in females, but not in males, and vice versa dihydrotestosterone in males, but not in females.

In this study, we found more large spines (head diameter ≥ 0.6 μm) in females, independent of the estrus cycle, than in males, and consistently a higher expression of NMDA receptor expression in females than in males. Furthermore, similar results, both in vibratome sections of adults and P0 mice and in perinatal cultures, strongly suggest that differences between male and female hippocampal neurons in adults result from sex-specific differentiation processes during development.

## Results

### Spine type density in male and female Thy1-GFP mice

**Figure 1:**
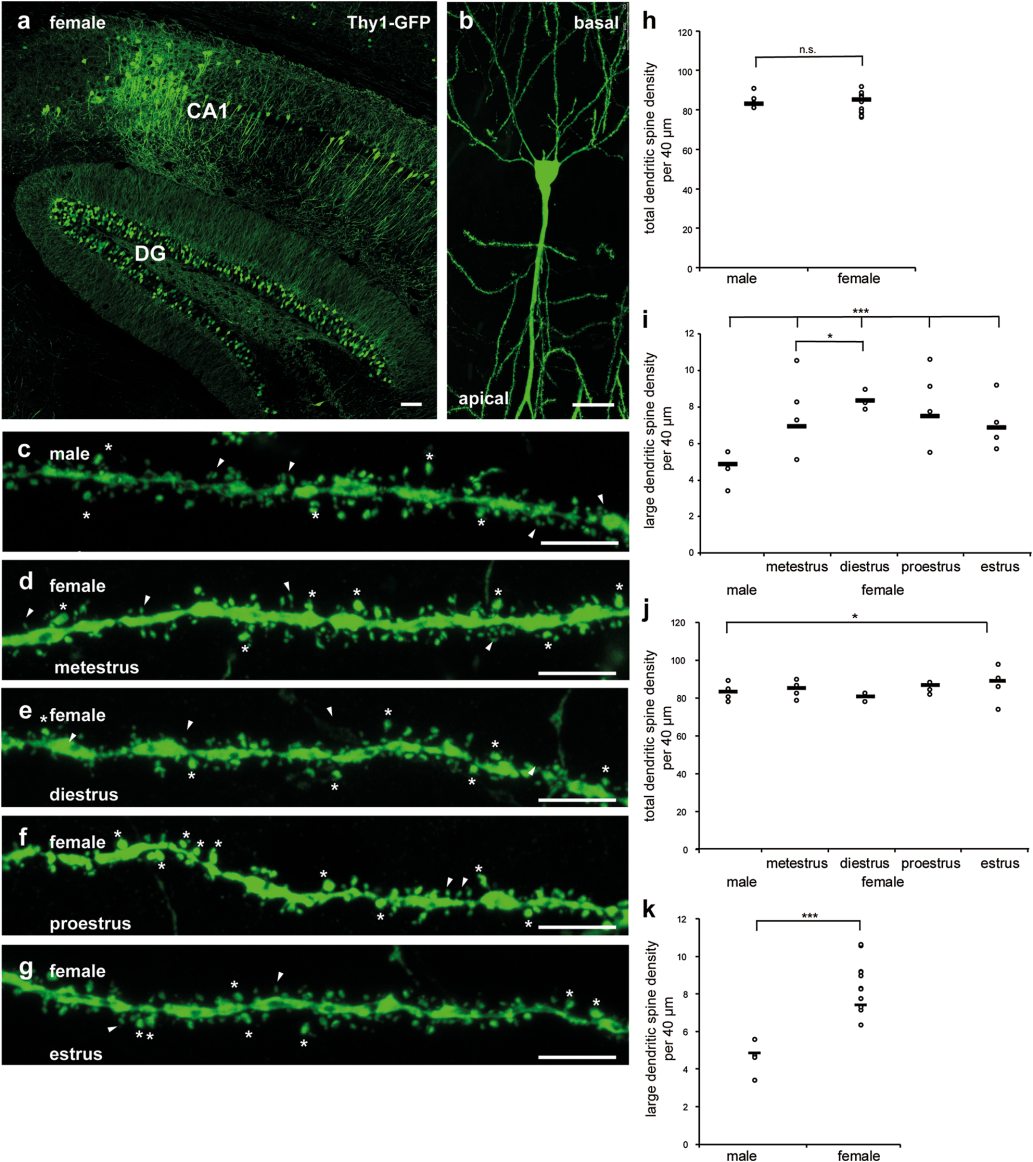
Hippocampal section of the Thy1-GFP mouse showing sparse expression of GFP in isolated pyramidal neurons and granule cells. Female hippocampal neurons display more large spines than male neurons in vibratome sections of hippocampal tissue. (**a**) A representative section (confocal stack) of adult female Thy1-GFP mouse hippocampus was stained with an antibody to GFP to enhance the GFP immunolabeling and visualize the morphology. Scale bar represents 75 µm. Abbreviations: CA1 (cornu ammonis), DG (dentate gyrus). (**b**) Representative example of a female pyramidal neuron (confocal stack) from the hippocampal CA1 region expressing a genetically encoded GFP, allowing for analyzing spine morphology in vibratome sections of hippocampal tissue. Scale bar represents 25 µm. Confocal image (stack) of a (**c**) male, (**d**) female metestrus, (**e**) female diestrus, (**f**) female proestrus and a (**g**) female estrus CA1 hippocampal dendrite with large (indicated with asterisks) and other (indicated with arrow heads) spines are shown. At this magnification individual spines can readily be classified. Scale bar represents 5 µm. (**h**) Histogram showing the total apical dendritic spine density after three-dimensional reconstruction of male and female neurons. In the scatterplot “**−**” indicates the mean. Quantification of spine densities in hippocampal sections of EGFP-Thy1 mice reveals no difference in total apical spine number (p = 0.245; mixed model analysis; K–S test; n = 4 animals per male and n = 4 per cycle stage (n = 12 in total) with at least 240 dendrites (male) and 463 dendrites (female) in total). (**i**) Quantification of spine densities reveals an increase in apical large spine number in all four cyclic stages of female mice in hippocampal sections of EGFP-Thy1 mice (***p ≤ 0.0001; mixed model analysis; K–S test) as compared to male mice, as well as an increase in diestrus female animals as compared to metestrus female animals (*p = 0.05; mixed model analysis; K–S test; n = 4 animals per male and n = 4 per cycle stage with at least 120 dendrites (male) and 61 dendrites (female metestrus), 50 dendrites (female diestrus), 60 dendrites (female proestrus), 62 dendrites (female estrus) in total). In the scatterplot “**−**” indicates the mean. (**j**) Quantification of spine densities reveals no difference in total apical spine number except for male versus female estrus stage (*p = 0.021; mixed model analysis; K–S test; n = 4 animals per male and n = 12 per female (n = 4 per cycle stage) with at least 240 dendrites (male) and 463 dendrites (female) in total). In the scatterplot “**−**” indicates the mean. (**k**) Quantification of spine densities reveals an increase in apical large spine number in female mice in hippocampal vibratome sections of EGFP-Thy1 mice (***p ≤ 0.0001; mixed model analysis; K–S test; n = 4 animals per male and n = 4 per cycle stage (n = 12 in total) with at least 120 dendrites (male) and 233 dendrites (female) in total). In the scatterplot “**−**” indicates the mean.

We determined spine density on apical dendrites of hippocampal neurons in the CA1 region of Thy1-GFP mice. In hippocampal vibratome sections of these transgenic mice only single neurons were stained, and the dendritic spines along their dendrites were clearly discernible (Fig. 1a–g). We found all types of spines mentioned by Sorra and Harris^32^: filopodia, which are considered to represent immature spines; thin spines with a total length greater than the neck diameter ending in a small bulbous head; short and wide stubby spines with no constriction in their neck; sessile spines, which are longer than their diameter, with no bulbous head, and mushroom spines, with a constricted neck and a large irregular head.

Surprisingly, the total numerical density of spines per 40 µm length of dendrite was almost identical in the neurons of male and female animals, as long as we used the average value of all cyclic stages in females (Fig. 1h) (means ± SD for numerical density of spines per 40 µm length of dendrite examined by mixed model analysis; total spines: male: 85.507 ± 1.224; female: 85.322 ± 0.891; [F(1/92.502) = 0.015; p = 0.903]; post-hoc analysis variances between groups [F(1/92.502) = 0.015; p = 0.903] statistical power (1 − ß err prob) 6,9%; n = 4 animals per male and n = 4 per cycle stage (n = 12 in total) with at least 240 dendrites (male) and 463 dendrites (female) in total; K–S test p = 0.200). Taking estrus cyclicity into account, thus comparing single values of the estrus stages, we however observed that the number of large spines (head diameter ≥ 0.6 μm) was lower in male animals than in female animals at each stage of the cycle (Fig. 1i) (means ± SD for numerical density of spines per 40 µm length of dendrite examined by mixed model analysis; large spines: male: 4.87 ± 0.418; female metestrus: 6.935 ± 0.595; female diestrus 8.365 ± 0.633; female proestrus 7.492 ± 0.577; female estrus 6.881 ± 0.573 [F(4/24.211) = 18.243; p ≤ 0.0001]; post-hoc analysis variances between groups [F(4/24.211) = 18.243; p ≤ 0.0001] statistical power (1 − ß err prob) 100%; n = 4 animals per male and n = 4 per cycle stage with at least 120 dendrites (male) and 61 dendrites (female metestrus), 50 dendrites (female diestrus), 60 dendrites (female proestrus), 62 dendrites (female estrus) in total; K–S test p ≤ 0.0001). In contrast to findings in the rat^18^, the density of all spines did not vary with the estrus cycle in mice (Fig. 1j) (means ± SD for numerical density of spines per 40 µm length of dendrite examined by mixed model analysis; total spines: male: 83.211 ± 1.490; female: 85.343 ± 1.060; [F(1/178.440) = 1.360; p = 0.245]; post-hoc analysis variances between groups [F(1/178.440) = 1.360; p = 0.245] statistical power (1 − ß err prob) 0.308; n = 4 animals per male and n = 12 per female (n = 4 per cycle stage) with at least 240 dendrites (male) and 463 dendrites (female) in total; K-S test p ≤ 0.0001).

Changes related to the estrus cycle were only seen in large spines (Fig. 1i). The density of large spines was highest during diestrus, when estradiol levels increase (Fig. 1i). No difference was seen in total spine number except for in males, and during the estrus stage in females (Fig. 1j).

As a final result, determination of spine type density in apical dendrites demonstrated a clear sex-dependency. We found that in females the average number of large spines, defined by a head diameter ≥ 0.6 μm, was higher than in males, even at each stage of the estrus cycle, despite the fact that the number of large spines varied during the cycle. When all estrus cyclic stages were considered, the number of large spines was ≈ 34% higher as compared to the number of large spines in male animals (Fig. 1k) (means ± SD for numerical density of spines per 40 µm length of dendrite examined by mixed model analysis; large spines: male: 4.87 ± 0.418; female: 7.418 ± 0.297; [F(1/187.384) = 42.257; p ≤ 0.0001]; post-hoc analysis variances between groups [F(1/187.384) = 42.257; p ≤ 0.0001] statistical power (1-ß err prob) 100%; n = 4 animals per male and n = 4 per cycle stage (n = 12 in total) with at least 120 dendrites (male) and 233 dendrites (female) in total; K–S test p ≤ 0.0001).

**Figure 2:**
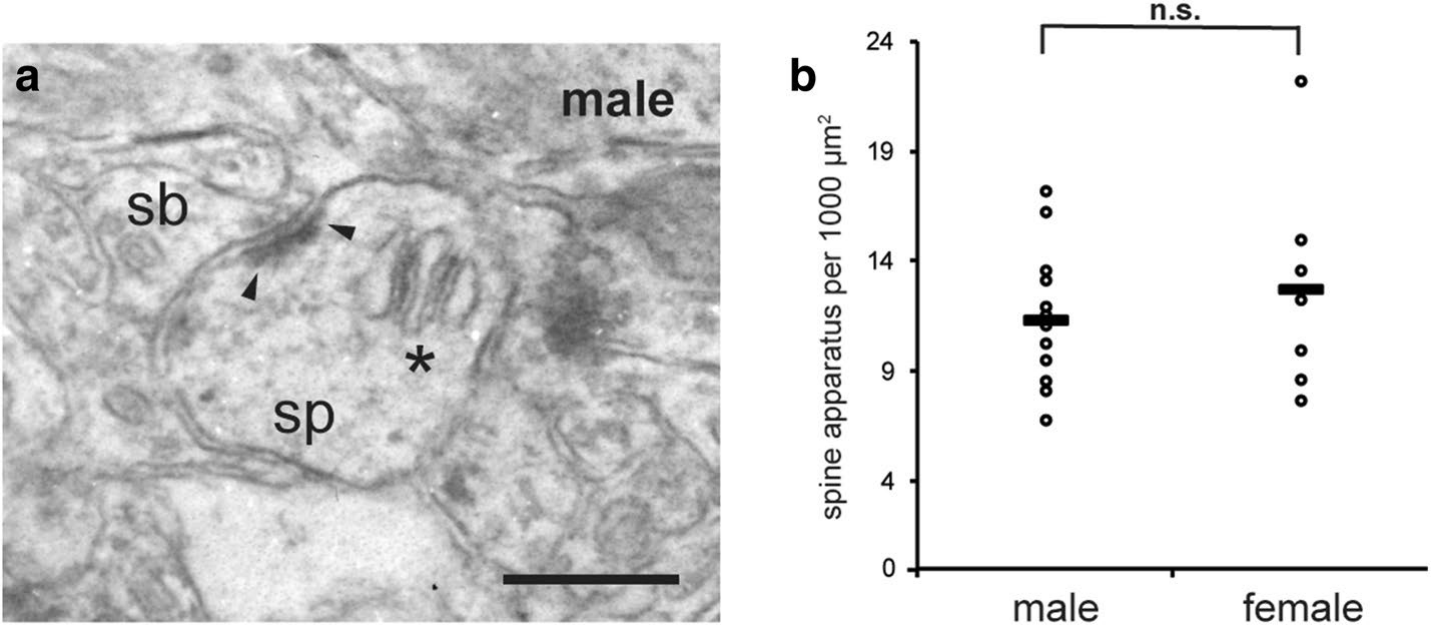
The number of spine apparatuses is similar in the hippocampus of male and female wild-type (WT) mice in situ*.* (**a**) Representative example of a spine apparatus (SA, asterisks) in the neck of a dendritic spine (sp) in stratum radiatum of CA1 region of the hippocampus of male WT mice; postsynaptic density (arrow heads), synaptic bouton (sb). Scale bar represents 250 nm. (**b**) Counts of spine apparatuses revealed no difference in spine apparatus number in male and female WT (p = 0.484; mixed model analysis; n = 4 male and n = 3 female animals). In the scatterplot “**-**” indicates the median.

Remarkably, the number of spine apparatuses did not differ between male and female animals (Fig. 2a,b) (means ± SEM for spine apparatus per 1,000 µm^2^ in stratum radiatum of CA1: male: 11.51 ± 0.91, n = 4; female: 12.99 ± 1.44, n = 3; mixed model, p = 0.484). According to Spacek and Harris^14^, 80% of large spines contain a spine apparatus; thus at first glance it appears that the difference of 20% between males and females in the number of large spines concerns spines that do not contain a spine apparatus. To verify this hypothesis, however, an extensive EM analysis would be required.

### NMDA receptor expression in male and female mice

**Figure 3:**
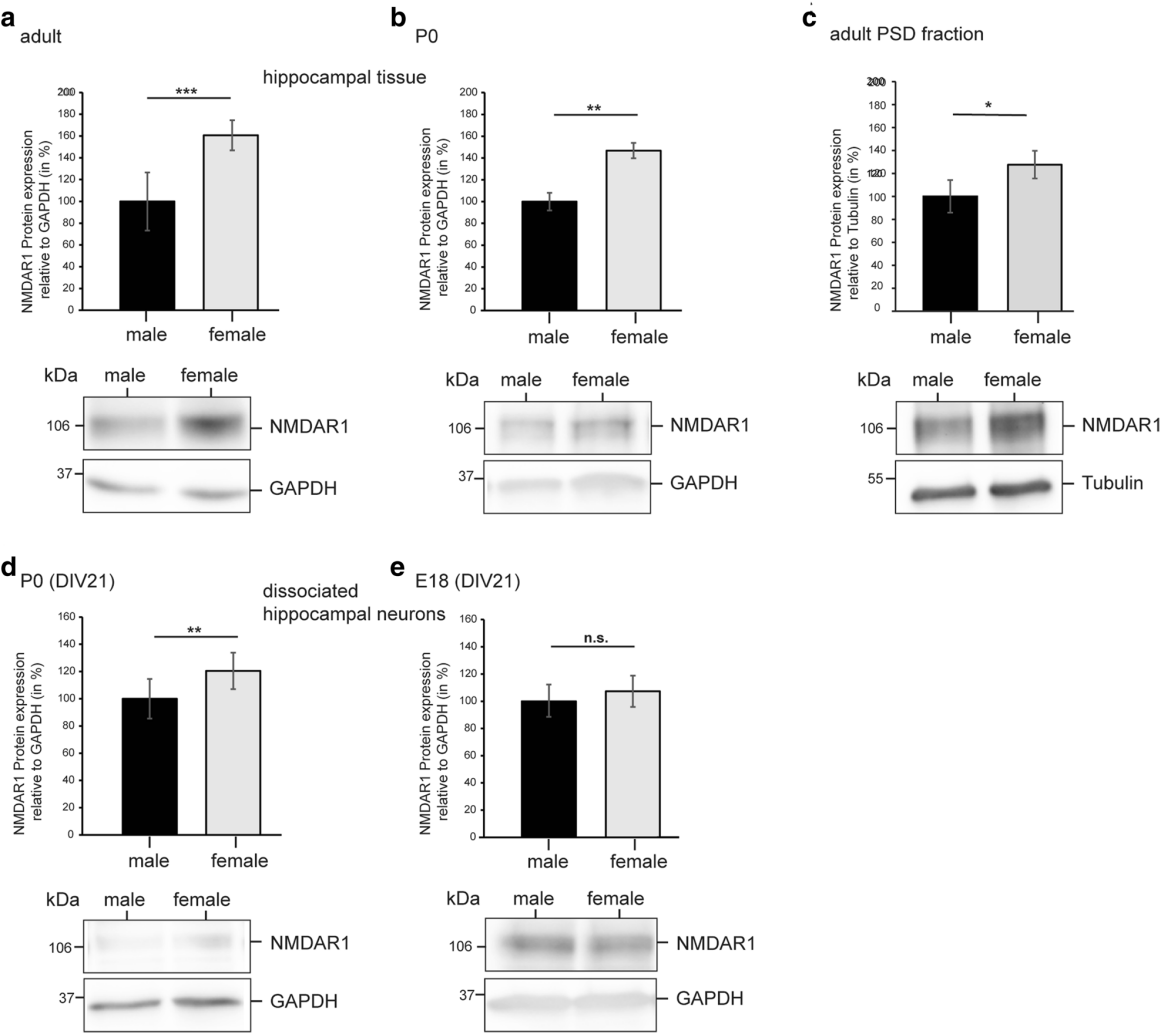
Female hippocampal neurons display more NMDAR1 receptors than male neurons. Western blot analyses of endogenous NMDAR1 levels in hippocampal tissue and cultured hippocampal neurons. All immunoblots were probed with either anti-NMDAR1 antibody (~ 106 kDa) or anti-GAPDH monoclonal antibody (loading control; ~ 37 kDa). Protein levels were quantified and normalized according to the levels of GAPDH protein. Abbreviations: P0 (postnatal day 0), E18 (embryonic day 18), DIV (days in vitro). Please note that different exposure times are shown. Data are normalized to male levels. (**a**) Quantitative analysis of hippocampal tissue of adult male and female mice revealed stronger expression of NMDAR1 in female mice (***p < 0.001; Mann–Whitney U test; male: n = 6 animals with n = 10 blots, female: n = 8 animals with n = 10 blots). (**b**) Quantitative analysis of hippocampal tissue of P0 male and female mice revealed stronger expression of NMDAR1 in female mice (**p = 0.007; Mann–Whitney *U* test; male: n = 10 animals with n = 8 blots, female: n = 8 animals with n = 8 blots). (**c**) The adult female hippocampal PSD fraction displays more NMDAR1 receptors than the male hippocampal PSD fraction. Western blot analyses of endogenous NMDAR1 levels in hippocampal PSD fraction. All immunoblots were probed either with anti-NMDAR1 antibody (~ 106 kDa) or anti-Tubulin monoclonal antibody (loading control; ~ 55 kDa). Protein levels were quantified and normalized according to the levels of Tubulin protein (*p = 0.05; Student’s t-test; male: n = 14 animals used for PSD preparation with n = 6 blots, female: n = 15 animals used for preparation with n = 6 blots). (**d**) Quantitative analysis of male and female hippocampal neurons at DIV21 (preparation day P0) revealed stronger expression of NMDAR1 in female cultures (**p = 0.006; Mann–Whitney U test; n = 4 independent cultures per sex with n = 12 blots). (**e**) No difference in NMDAR1 expression was observed after quantitative analysis of male and female hippocampal neurons after 21 days in culture (DIV21; preparation day E18; p = 0.492; Mann–Whitney *U* test; n = 4 independent cultures per sex with n = 11 blots).

**Figure 4:**
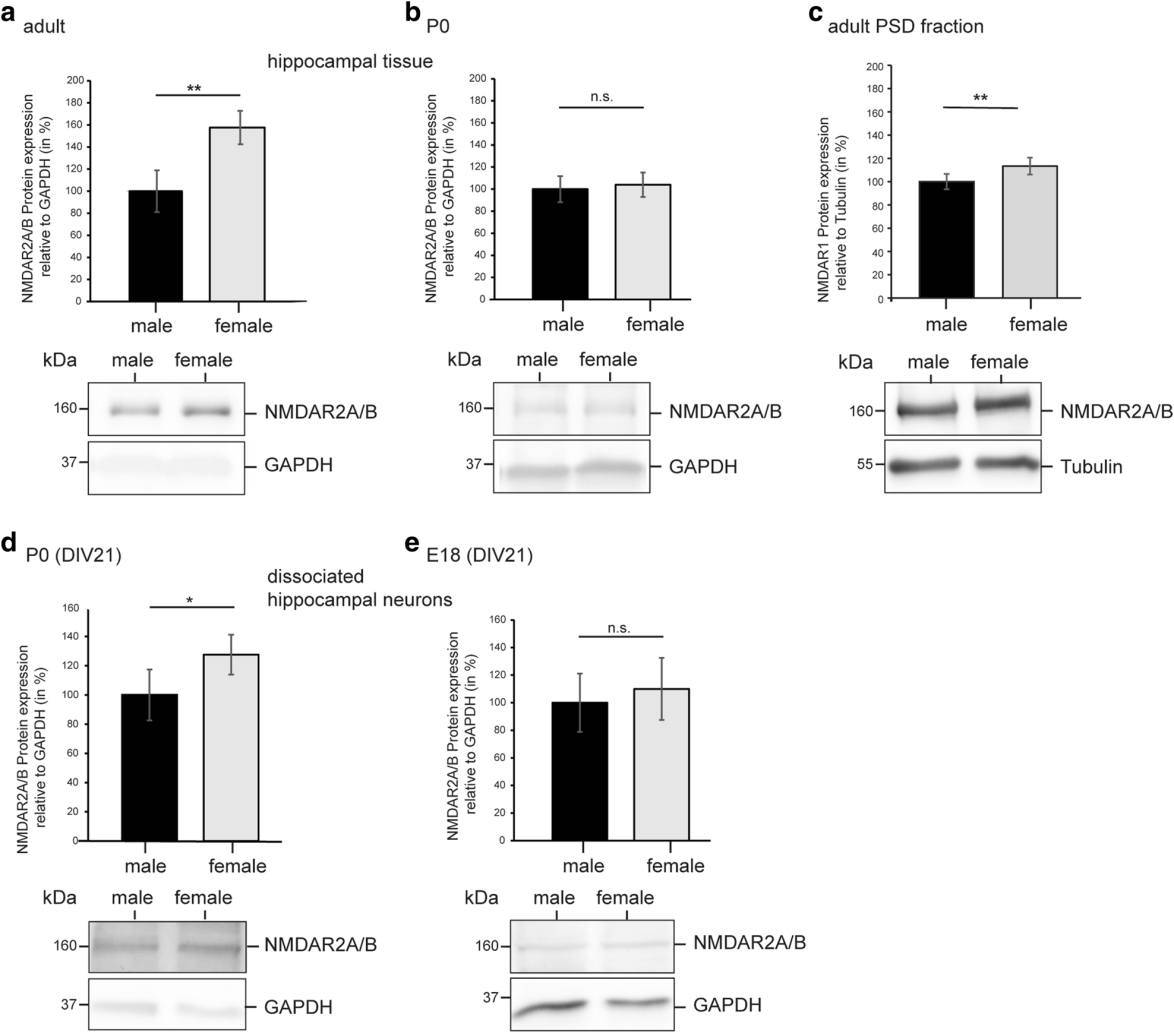
Western blot analyses of endogenous NMDAR2A/B levels in hippocampal tissue or of cultured hippocampal neurons. All immunoblots were probed with either anti-NMDAR2A/B antibody (~ 160 kDa) or anti-GAPDH monoclonal antibody (loading control; ~ 37 kDa). Protein levels were quantified and normalized according to the levels of GAPDH protein. Abbreviations: P0 (postnatal day 0), E18 (embryonic day 18), DIV (days in vitro). Please note that different exposure times are shown. Data are normalized to male levels. (**a**) Quantitative analysis of hippocampal tissue of adult male and female mice revealed stronger expression of NMDAR2A/B in female mice (**p = 0.002; Mann–Whitney *U* test; male: n = 6 animals with n = 9 blots, female: n = 8 animals with n = 9 blots). (**b**) Quantitative analysis of P0 hippocampal tissue of male and female mice revealed no difference in NMDAR2A/B expression (p = 0.419; Mann–Whitney *U* test; male: n = 10 animals with n = 10 blots, female: n = 8 animals with n = 10 blots). (**c**) The adult female hippocampal PSD fraction displays more NMDAR2A/B receptors than the male hippocampal PSD fraction. Western blot analyses of endogenous NMDAR2A/B levels in hippocampal PSD fraction. All immunoblots were probed either with anti-NMDAR2A/B antibody (~ 160 kDa) or anti-Tubulin monoclonal antibody (loading control; ~ 55 kDa). Protein levels were quantified and normalized according to the levels of Tubulin protein (**p = 0.014; Student’s t-test; male: n = 14 animals used for PSD preparation with n = 7 blots, female: n = 15 animals used for PSD preparation with n = 7 blots). (**d**) Quantitative analysis of male and female hippocampal neurons at DIV21 (preparation day P0) revealed stronger expression in NMDAR2A/B expression in hippocampal cultures (*p = 0.015; Mann–Whitney *U* test; n = 4 independent cultures per sex with n = 8 blots). (**e**) No significant difference in NMDAR2A/B expression was observed in male and female hippocampal neurons in culture at DIV21 (preparation day E18 p = 0.369; Mann–Whitney *U* test; n = 4 independent cultures per sex with n = 8 blots).

Since large spines have larger postsynaptic densities, which anchor more NMDA and AMPA receptors than small spines, we hypothesized that the expression of NMDA and AMPA receptors should be similarly sex-dependent. For these experiments, we used randomly chosen adult animals and ignored the estrus stage, since the number of large spines was significantly higher at each stage of the estrus cycle. In fact, when we studied the expression of NMDA-R1 and NMDA-R2A/B by Western blot analysis we found a clear-cut difference after quantitative evaluation of the blots using image analysis. With both NMDA-R1 and NMDA-R2A/B, the expression was more than 50% higher in adult hippocampal tissue of wild-type females than in adult males (Figs. 3a, 4a) (NMDA-R1: ***p ≤ 0.001 for adult male and female animals, means ± SEM%: male: 100 ± 26.7, female: 160.7 ± 13.9; male: n = 6 animals with n = 10 blots, female: n = 8 animals with n = 10 blots; NMDA-R2A/B: **p = 0.002 for adult male and female animals, means ± SEM%: male: 100 ± 18.9, female: 157.6 ± 15.2; male: n = 6 animals with n = 9 blots, female: n = 8 animals with n = 9 blots). To rule out extrasynaptic NMDA receptors, we also tested our hypothesis in fractions of postsynaptic densities (PSD). Similarly, in PSD fractions from hippocampal tissue sex-dependency is also obvious (Figs. 3c, 4c) (NMDA-R1: *p = 0.05 for adult male and female PSD fraction of animals, means ± SEM%: male: 100 ± 14.2, female: 127.6 ± 12; male: n = 14 animals used for PSD preparation with n = 6 blots, female: n = 15 animals used for preparation with n = 6 blots; NMDA-R2A/B: **p = 0.014 for adult male and female PSD fraction of animals, means ± SEM%: male: 100 ± 6.6, female: 113.3 ± 7.3; male: n = 14 animals used for PSD preparation with n = 7 blots, female: n = 15 animals used for PSD preparation with n = 7 blots).

As a next step, we wanted to find out whether the differences between sexes were also seen shortly after birth (P0). A sex difference at this point of time could point to sex-specific differentiation, which might be induced during the perinatal period, when sexual imprinting takes place due to high testosterone levels in males. The sex-dependent difference between males and females was also seen in hippocampal tissue of newborn animals with respect to NMDA-R1 expression (Fig. 3b). However, this was not the case regarding NMDA-R2A/B expression (Fig. 4b) (NMDA-R1: **p = 0.007 for P0 male and female animals, means ± SEM%: male: 100 ± 8.1, female: 146.8 ± 7.01; male: n = 10 animals with n = 8 blots, female: n = 8 animals with n = 8 blots; NMDA-R2A/B: p = 0.419 for P0 male and female animals, means ± SEM%: male: 100 ± 11.8, female: 104 ± 11.1; male: n = 10 animals with n = 10 blots, female: n = 8 animals with n = 10 blots).

In a further attempt to approach sex-specific differentiation, we performed Western blots from dissociated hippocampal cultures (Figs. 3d, e, 4d,e). These cultures have the advantage that all influences of gonadal sex steroid hormones are excluded in these experiments. We used rats for these experiments, as sex-dependent differences in spine type density had previously been shown in cultures of rats^29^. In addition, cultures of rats are more easily generated and more stable than cultures from mice. Hippocampal neurons prepared from postnatal day 0 (P0) and embryonic day 18 (E18) male and female animals were used, and the dissociated neurons were cultivated for three weeks. We hypothesized that the comparison of these cultures from different ages would allow us to find out whether the perinatal surge of testosterone affects the expression level of NMDA receptors. At the end of the culture period of three weeks, we found no sex-specific difference in NMDA-R1 and NMDA-R2A/B expression in cultures prepared from E18 animals (Figs. 3e, 4e) (NMDA-R1: p = 0.492 for E18 male and female cultures, means ± SEM%: male: 100 ± 12.3, female: 107.3 ± 11.5; n = 4 independent cultures per sex with n = 11 blots; NMDA-R2A/B: p = 0.369 for E18 male and female cultures, means ± SEM%: male: 100 ± 21.1, female: 110 ± 22.5; n = 4 independent cultures per sex with n = 8 blots). However, in contrast, after three weeks in culture a significant difference was found in cultures generated from P0 rats, suggesting that the perinatal surge of testosterone induces sex-specific differentiation (Figs. 3d, 4d) (NMDA-R1: **p = 0.006 for P0 male and female cultures, means ± SEM%: male: 100 ± 14,5, female: 120 ± 13,4; n = 4 independent cultures per sex with n = 12 blots; NMDA-R2A/B: *p = 0.015 for P0 male and female cultures, means ± SEM%: male: 100 ± 21,3, female: 126,6 ± 17,4; n = 4 independent cultures per sex with n = 8 blots). E18 pups were not exposed to the testosterone surge, and accordingly, no difference was seen between cultures from male and female animals. As in adults, no sex difference could be found in both cultures deriving from E18 or P0 with respect to GluR1 expression (data not shown).

## Discussion

Our study reveals that in the CA1 hippocampal region the density of large spines along dendrites of pyramidal neurons is higher in female animals than in male animals, as previously shown in dissociated hippocampal cultures^29,31^. In addition, the expression of NMDA-R1 and NMDA-R2A/B is stronger in hippocampi of adult female animals than in those of male animals. The comparison of hippocampal cultures of E18 and P0 male and female animals points to sex-specific differentiation processes during the perinatal period. Cultures of male and female pups generated before birth (E18) showed no differences, while cultures generated after birth (P0) revealed a clear sex-dependency.

Our counts of total spine densities confirm previous data in rats^33–35^. The average numbers of spines during the estrus cycle are in the range of total average spine numbers in rats. Estrus cyclicity, however, was less pronounced in mice compared to rats^18,21^ and was only clearly apparent in the group of large spines. A study by Li et al.^36^ also used the Thy1-GFP mouse to calculate spine numbers in males. Our data confirm their counts of total spines in the stratum radiatum of the CA1 hippocampal region.

In rats, it has long been known that spine and spine synapse density vary during the estrus cycle and it is highest at proestrus, when estradiol levels are at their maximum^18,21^. Strikingly, similar data on estrus cyclic dependency of spine density in mice have not yet been published, presumably because estrus cyclicity is not seen as long as all spine types are concerned Only Spencer and coworkers^37^ have studied the expression of various proteins at specific stages of the estrus cycle in mice, and they found that cycle-dependent expression of synaptic proteins, such as PSD95, is highly expressed at proestrus. Since PSD95 is typically expressed in postsynaptic densities, which are larger in large spines than in all other spine types, cyclicity of PSD95 expression in mice is consistent with our finding that estrus cyclicity only concerns large spines in mice. Nevertheless, it appears that cyclicity of spine density exists in mice, as in rats. In an earlier study by the McEwen group^38^, they found in mice, in contrast to rats, no increase in spines in response to estradiol treatment of ovariectomized mice. They observed, however, an increase in the number of mushroom spines upon estradiol treatment, which lead them to believe that the enlargement of the spine head is regulated by gonadal estradiol. It is of note that a new paradigm emerged by demonstrating that estradiol-induced synaptic plasticity in the hippocampus is unlikely to rely on gonadal estradiol, but that estrogenic effects are induced by local synthesis of estradiol, which is, in turn, regulated by Gonadotropin Releasing Hormone^21,23^. Downregulation of estradiol synthesis induced spine synapse loss in ovariectomized animals^39^ and in hippocampal slice cultures of female animals^20,29^.

A pivotal role of large spines in memory has frequently been shown^7^. Large spines with large heads are stable and contribute to strong synaptic connections^9,40^. Synapse maintenance over long periods is implicated in long-term memory, and the density of large spines correlates with memory performance^9,41^. Alzheimer’s disease, the most common reason for elderly dementia, is associated with the loss of predominantly large spines^42–46^. Reduction of large spines has also been reported in the context of schizophrenia^40^. Finally, lead exposure results in obvious deficits in the proportion and size of large spines, paralleled by a decrease in Arc/Arg3.1 expression, a protein strongly associated with memory^47^.

Interestingly, the number of spine apparatuses did not differ between sexes, although a spine apparatus is a typical feature of large spines. As large spine density is not altered in the synaptopodin knock-out mouse, which lack a spine apparatus^17^, one may conclude that, at least under control conditions, the formation of a spine apparatus is independent of the formation of large spines. Synaptopodin, however, the protein which appears to be required for the formation of spine apparatuses, is more strongly expressed in females than in males, similar to the differences in large spine density between males and females seen in our study^48^.

Consistent with our data on large spine density, we found that the expression of NMDA-R subtype1 and the NMDA-R type 2A/B is significantly stronger in adult females than in males. Sex differences in baseline expression of NMDA-R1 and NMDA-R2A/B have previously been shown in rats^49–54^, thus confirming our data. Nevertheless, there are discrepancies, which are likely due to the cyclic phase of the animals during testing^45,55^. It has been demonstrated that male rats express higher levels of NMDA receptors in the hippocampus than females only during the estrus stage, while females in the diestrus phase show higher NMDA receptor densities than during the estrus phase^56^.

Sex-dependent differences in the induction of LTP, which highly depends on NMDA receptor activation, were also reported^57–60^. In rats of both sexes, Montfort and coworkers found that sex does not affect the amount of GluR1 in the hippocampus. They found, however, that the magnitude of LTP was significantly lower in females than in males. In this study, however, randomly chosen female rats were used, and it needs to be taken into account that considerable differences in LTP induction exist during the estrus cycle, as has previously been shown in mice^61^. We found that in mice the average potentiation after tetanizing stimulation was similar in males and females^62,63^.

Theta-burst stimulation to induce LTP leads to the activation of NMDA receptors, which induces translocation of AMPA receptors to the synaptic membrane. LTP is the enduring enhancement of synaptic transmission and is thought to be the cellular correlate of learning and memory. In view of our results on sex-specific NMDA receptor expression, together with the lack of differences in the LTP magnitude^62^, LTP induction appears not to depend on the degree of NMDA receptor expression.

Similar results in hippocampal cultures and in animals, as far as large spines^29,62^ and NMDA receptors are concerned, strongly suggest that differences in spine type density between males and females do not result from differences in gonadal sex steroid production in adults. From our data, particularly on NMDA receptor expression, it would appear that the differences between sexes result from perinatal sexual differentiation between E18 and P0. NMDA-R1, as well as NMDA-R2A/B expression, in hippocampal cultures of P0 animals after three weeks in culture, is higher in “female” than in “male” cultures, similar to that in adult animals, while such a difference was not found in cultures of E18 animals. Thus, differences in hippocampal circuitry are very likely acquired during the critical perinatal period. Even if in the case of NMDA-R2A/B no differences were seen in newborn mice, the differences seem to develop postnatally. The question as to whether sex steroid synthesis in the neurons participate in this process, as recently shown for estradiol in neuritogenesis, remains to elucidated^64^.

## Material and methods

### Animals

Wistar rats and C57BL/6 mice (Institute of Neuroanatomy, University of Hamburg, Germany) were maintained under controlled conditions, and water and food were available ad libitum. All experiments were carried out in accordance with the institutional guidelines for animal welfare and approved by the “Amt für Verbraucherschutz, Lebensmittelsicherheit und Veterinärwesen” (Freie und Hansestadt Hamburg; approval ID: ORG735, ORG880, ORG728, ORG604, ORG850). Embryonic day 18 (E18) and postnatal day 0 (P0) rats were used for culturing hippocampal cells in dispersion. Young postnatal mice (P0) and adult mice (7–10 weeks old) were decapitated, and the hippocampi were dissected for protein preparation or postsynaptic density preparation (PSD).

The adult Thy1-GFP mice aged eight months (n = 4 male and n = 12 female) used for these experiment were kindly provided by Dr. B. Brunne and Prof. M. Frotscher (Center of Molecular Neuroscience (ZMNH), University of Hamburg, Germany)^30^. Estrus cyclicity in female mice was determined by vaginal smears, which were stained by the Pappenheim method (see Supplementary Material). Staging was performed postmortem. Mice at metestrus, diestrus, proestrus and estrus stages were used. The perfusion of the animals (4% paraformaldehyde in phosphate buffered saline) was already performed at the ZMNH. After perfusion, the brains were removed and postfixed in freshly prepared 4% paraformaldehyde (PFA) in phosphate-buffered saline (1 × PBS). The hemispheres were cut into 50 µm serial coronar sections on a vibratome. The sections were further processed for immunohistochemical staining (see below).

### Dispersed cultures

Primary hippocampal neurons were prepared from embryonic day 18–19 Wistar rats, as described by Banker and Goslin^65^. Neurons from P0 rats were dissociated in papain (Sigma-Aldrich, Taufkirchen, Germany). The cells were cultured on poly-l-lysine-coated (0.1 mg/ml) 6 well-plates in Neurobasal A medium, supplemented with 0.5% B27 (Life Technologies, St Paul, MN, USA), 0.5 mM glutamine (Life Technologies) and the antibiotics penicillin and streptomycin (1x, Life Technologies) at a density of 450.000 cells per well (6-well-plate). The purity of the neuronal culture was tested by immunohistochemistry according to Schassen et al.^66^. The cultures consisted of approximately 80% neuronal cells.

### Immunohistochemistry

Immunohistochemistry was performed on free-floating vibratome sections of adult Thy1-GFP hippocampi (n = 4 male and n = 12 female) to better visualize GFP fluorescence. After several brief rinses in 1 × PBS, vibratome sections were pre-incubated in blocking solution [1 × PBS containing 10% NGS (normal goat serum (Sigma-Aldrich))/0.5% Triton X-100 (T)] for 1 h at room-temperature with gentle agitation. After several brief washing steps, sections were incubated with a polyclonal antibody to GFP (Rb a GFP, ab 6556, Abcam, Cambridge, UK) (1:2,500) diluted in 3% NGS/PBS-0.5% T overnight at 4 °C. Sections were washed three times with 1 × PBS and incubated with the secondary antibody goat anti-Rabbit-IgG AlexaFluor 488 (Life Technologies) (1:500) diluted in 3% NGS/PBS-0.5% T for 1–2 h in the dark. After final washing steps (three times), the sections were mounted on glass slides, embedded in fluorescent mounting medium (Dako, Hamburg, Germany) and coverslipped. Sections were analyzed with a Leica SP5 confocal laser scanning microscope (Leica, Wetzlar, Germany).

### Lysate preparation and Western blot

The dissected hippocampi of P0 mice (C57BL/6 J), male (n = 10) and female (n = 8) were homogenized in 150 µl of homogenate buffer (RIPA: 150 mM NaCl, 50 mM Tris, 1% NP40 containing a protease inhibitor cocktail (Roche, Basel, Switzerland), followed by centrifugation at 20,000×*g* at 4 °C for 20 min, and the supernatant was collected and frozen for further use. For analysis of adult animals, the hippocampi of n = 6 male and n = 8 female mice (C57BL/6 J) were dissected and homogenized in a Mikro-Dismembranator in RIPA buffer containing 150 mM NaCl, 50 mM Tris, 1% NP40 and a protease inhibitor cocktail (Roche). The homogenates were centrifuged at 13,000×*g* at 4 °C for 30 min, and the supernatant was collected and frozen for further use. Protein concentrations were determined with the Bradford protein assay (Bio-Rad Laboratories, München, Germany). Hippocampal cell extracts, originating from male or female Wistar rats (E18 and P0) were prepared after 21 days in vitro (DIV21) with Tris-buffered saline (pH 7.4) containing 1.2% Triton X-100 and a protease inhibitor cocktail (Roche) (n = 4 cultures for each E18 and P0 cell extracts). Protein concentrations were determined with the Bradford protein assay (Bio-Rad). Extracts from adult hippocampi, P0 hippocampi and hippocampal cell culture were analyzed with a Western blot. After performing an SDS-PAGE with 8% acrylamide under reducing conditions, a subsequent Western blot (proteins were transferred to a nitrocellulose membrane) was treated 3% BSA (in PBS plus 0.3% Triton X-100) and analyzed with a monoclonal antibody to NMDAR1 (Mouse anti-NMDAR1, MAB363, Millipore, Billerica, MA, USA) (1:500), a polyclonal antibody to NMDAR2A/B (Rabbit anti-NMDAR2A/B, AB1548, Millipore) (1:500), and a monoclonal antibody to GAPDH (Mab anti-GAPDH, AM4300, Ambion, Kassel, Germany) (1:10,000). Incubation was for 12 h at 4 °C and 1 h at RT in the case of Mab anti-GAPDH. The secondary antibodies used were goat anti-mouse-IgG (1:2,500; Thermo Fisher Scientific, Dreieich, Germany) and donkey anti-rabbit-IgG (1:2,500; Thermo Fisher Scientific) conjugated to horseradish peroxidase. Incubation was for 1 h at room temperature. Proteins were detected using Immobilon Western Chemiluminescent HRP Substrate (Millipore) and quantified by densitometry using ImageJ (1.49n) software (means ± SEM; n = 1 refers to pooled neurons deriving from one culture).

PSD isolation was performed according to Carlin et al.^67^ with modifications. In brief, homogenization of hippocampi (male: n = 14, 7–10 weeks of age; female: n = 15, 8 weeks of age) was performed on ice in solution A (0.32 M sucrose, 4 mM Hepes pH 7.4, 1 mM MgCl_2_, 0.5 mM CaCl_2)_. After centrifugation at 1,400×*g* for 10 min at 4 °C the supernatant was discarded and the pellet resuspended and homogenized in solution A. A second centrifugation step was performed at 710×*g* for 10 min at 4 °C and the resulting supernatant was collected while the pellet was discarded. After a third centrifugation step at 13,800×*g* for 10 min at 4 °C, the supernatant contained the cytosolic fraction. The resulting pellet, containing the synaptosomes and mitochondria, was resuspended in solution B (0.32 M sucrose, 4 mM Hepes pH 7.4). The sucrose gradient consisting of 1.2 M Sucrose and 4 mM Hepes pH 7.4, 1.0 M Sucrose and 4 mM Hepes pH 7.4, and 0.85 M Sucrose and 4 mM Hepes pH 7.4 was overlaid with the resuspended pellet and centrifuged at 82,500×*g* for 2 h. The band between 1.0 and 1.2 M sucrose containing the synaptosomal fraction was removed and diluted with solution B and an equal volume of 1% Triton X-100, 0.32 M sucrose, 12 mM Tris pH 8.1. After stirring at 4 °C for 15 min, the suspension was centrifuged at 32,800×*g* for 30 min. The resulting pellets were resuspended in solution B, yielding the PSD fraction. 20 µg were used for immunoblotting. A monoclonal antibody to tubulin (Mouse anti-α-Tubulin, T6074, Sigma-Aldrich) (1:10,000) was used. Immunoblotting procedure was as described above.

### Electron microscopy

Electron microscopy was performed as described in^29^. Quantification of spine apparatuses was performed according to^17^. The percentage of spines with spine apparatus was determined in random ultrathin sections of hippocampus (stratum radiatum of CA1) from wild-type mice (male: n = 4; female: n = 3). A regular spine apparatus was considered to be present if at least two dense plates and at least two tubules of smooth ER were detected in close apposition.

### Image acquisition and data analysis

For quantification of dendritic spines, images were captured on a Leica SP5 confocal microscope with a 63 × objective and Z-series (z-step of 0.13 µm, a system optimized value which offered the best z-resolution and kept the amount of bleaching to a minimum) with 6 × zoom and 2048 × 512 pixels. Low magnification views were imaged with a 20 × objective and a z-step of 0.63 µm. To assess spine density and spine phenotype, a minimum of 90 cells for each group was randomly selected and 2–3 dendrites per neuron were analyzed to determine the ratio of large spines. Large spines were discriminated from other spine types by a bulbous-like head (head-diameter ≥ 0.6 µm; see^32^) (n = number of animals per sex (n = 4 animals per male and n = 12 animals per female (n = 4 per cyclic stage) with at least 117–463 dendrites). Spines on secondary and tertiary dendrites were measured and the distance to the soma varied from 50–100 µm (apical dendrites). Acquisition and data analysis were performed by investigators blind to the experimental conditions. All morphometric measurements were done with Neurolucida image analysis software.

### Statistics

All data are presented as means ± SEM or means ± SD. A level of significance of p ≤ 0.05 was adopted (*p ≤ 0.05, **p ≤ 0.01, ***p ≤ 0.001, n.s. = not significant). Spine data were analysed using a mixed model, after testing for normal distribution (Fig. 1h–k). In the case of Fig. 1i–k, values were converted via logarithmic transformation prior to statistical analysis. Data from Western Blots, quantified by densitometry using ImageJ (1.49n), were determined for each blot, and transformed into percentage of protein expression of female samples relative to male samples (which were set to a reference value of 100%). Afterwards, data from all blots could be compared. Percentage values were averaged, and depending on the data being parametric or nonparametric, were analyzed by either using a Student’s *t*-test or Mann–Whitney *U* test (Student’s *t*-test (parametric; paired, two-tailed): Figs. 3c, 4c; Mann–Whitney *U* test (nonparametric): Figs. 3a,b,d,e, 4a,b,d,e). Spine apparatus data were analysed using a mixed model (Fig. 2b). The statistical analysis was assessed with SPSS 24.0.0.2/26.0.

## Supplementary information

Supplementary information

## Data Availability

The data analyzed during the current study are available from the corresponding author on request.
