# Supplementary material for: Sex-specific features of spine densities in the hippocampus
Source: Sci Rep. 2020 Jul 9;10:11405. doi: 10.1038/s41598-020-68371-x (PMC7347548; doi:10.1038/s41598-020-68371-x)

Sex-specific features of spine densities in the hippocampus

Nicola Brandt[+], Tobias Löffler[+], Lars Fester, Gabriele M. Rune

Supplementary material 1

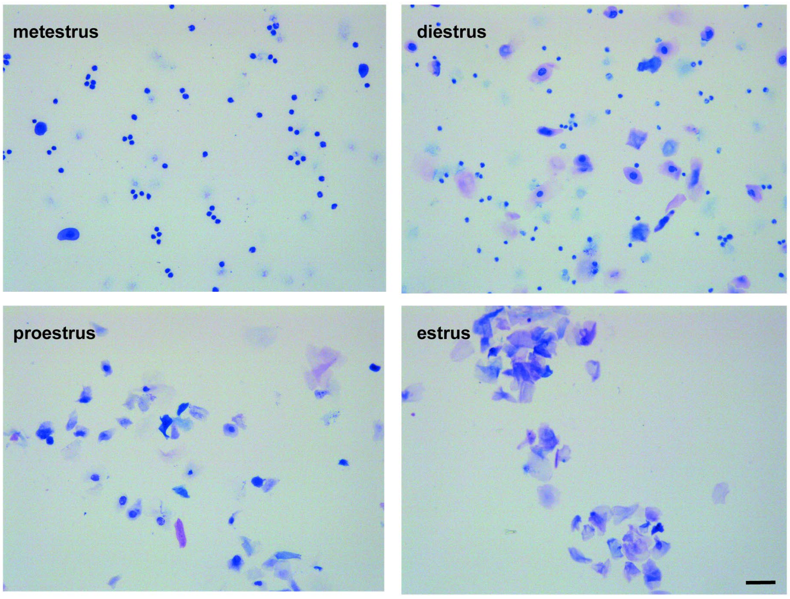

Supplementary figure 1: Estrus cyclicity in adult female Thy1-GFP mice.
Representative examples of the four stages of the estrus cycle in female Thy1-GFP mice showing the predominant cell typology in vaginal smears after staining with the Pappenheim method. Vaginal smear cytology reflects different estrus cyclic stages according to Byers et al., 2012. Images were captured on a Leica DM2000 LED Microscope with a 20x objective.
Scale bar represents 50 µm.

Supplemental data to Figure 3

a

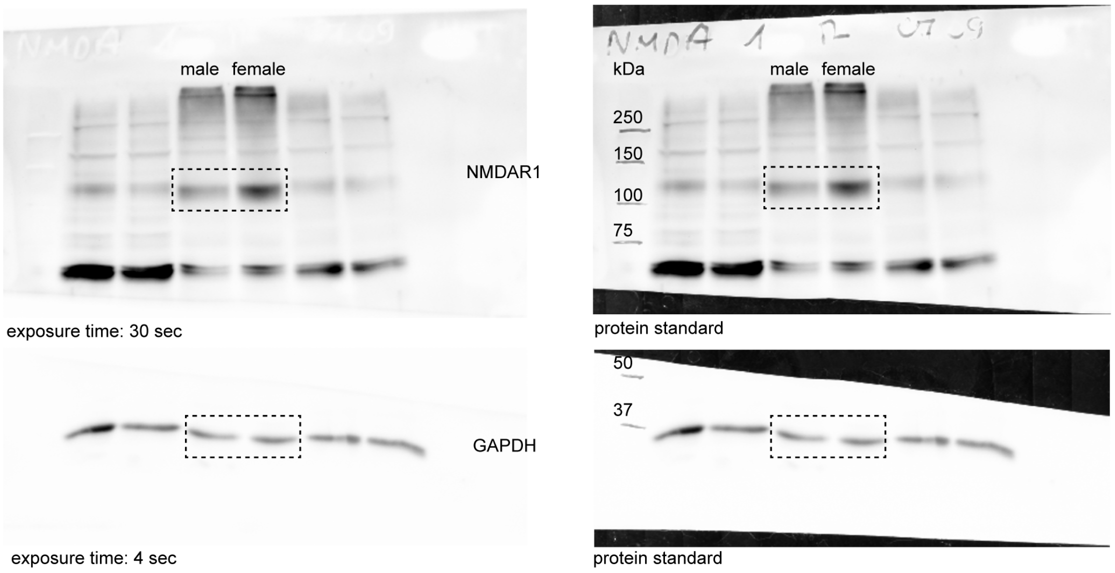

b

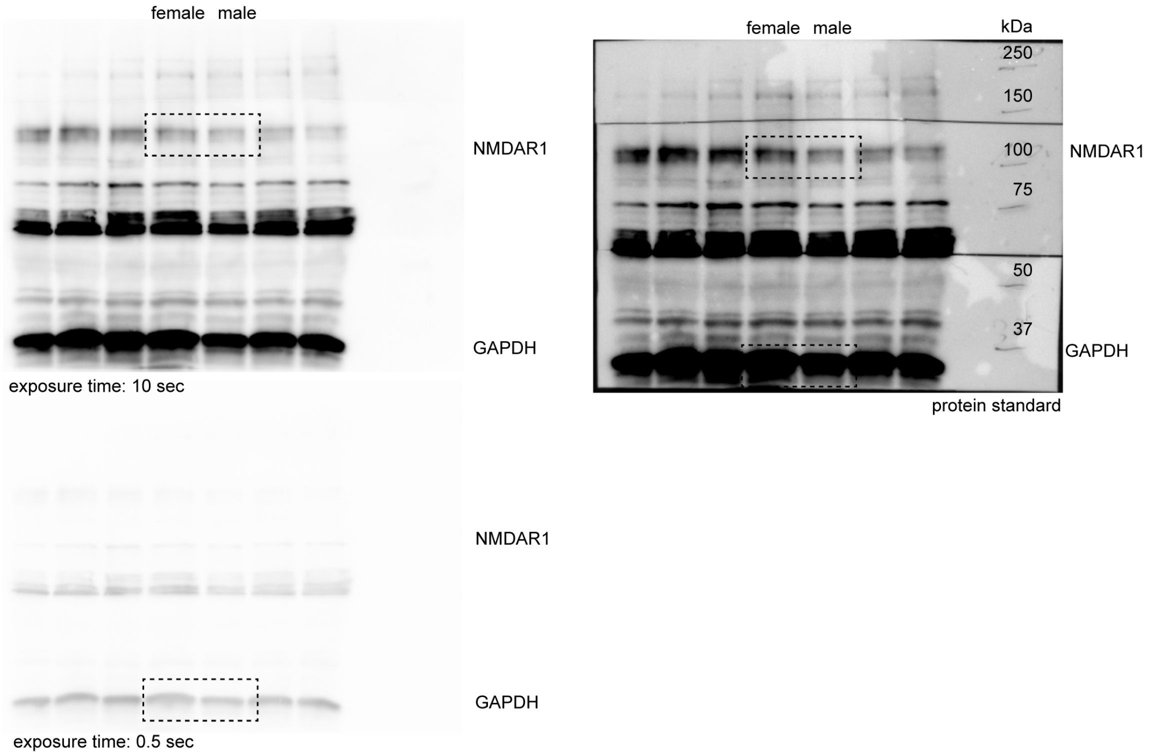

c

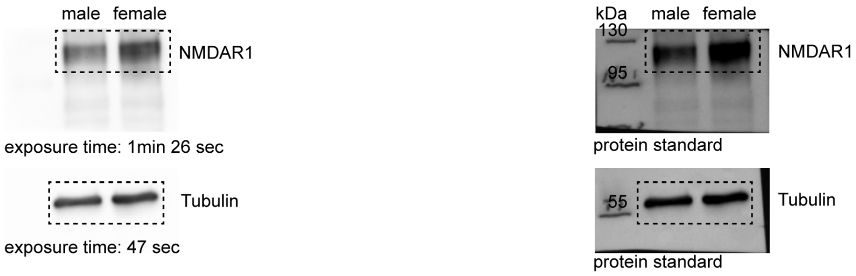

Supplemental data to Figure 3

d

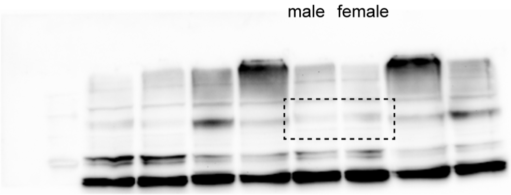

exposure time: 30 sec

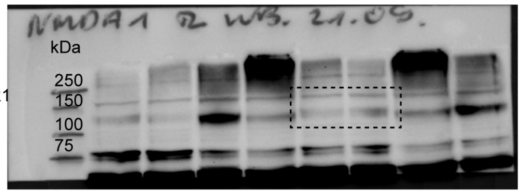

protein standard

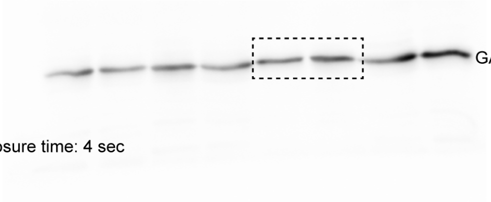

exposure time: 4 sec

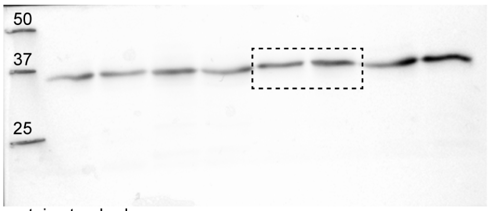

protein standard

e

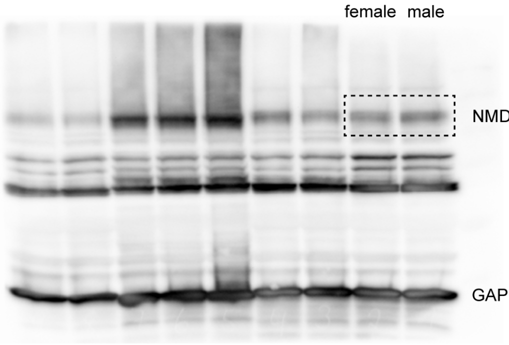

exposure time: 8 sec

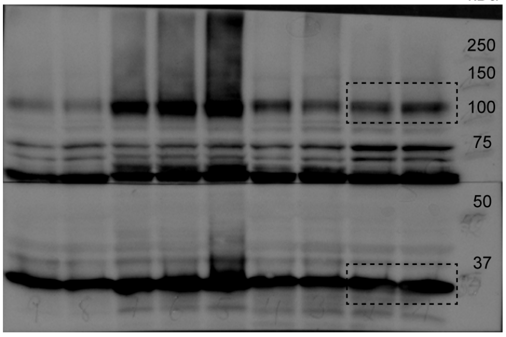

protein standard

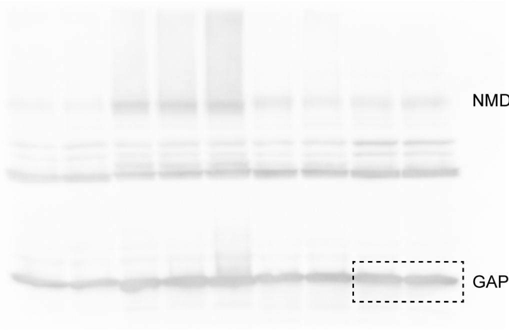

exposure time: 0.9 sec

Supplemental data to Figure 4

a

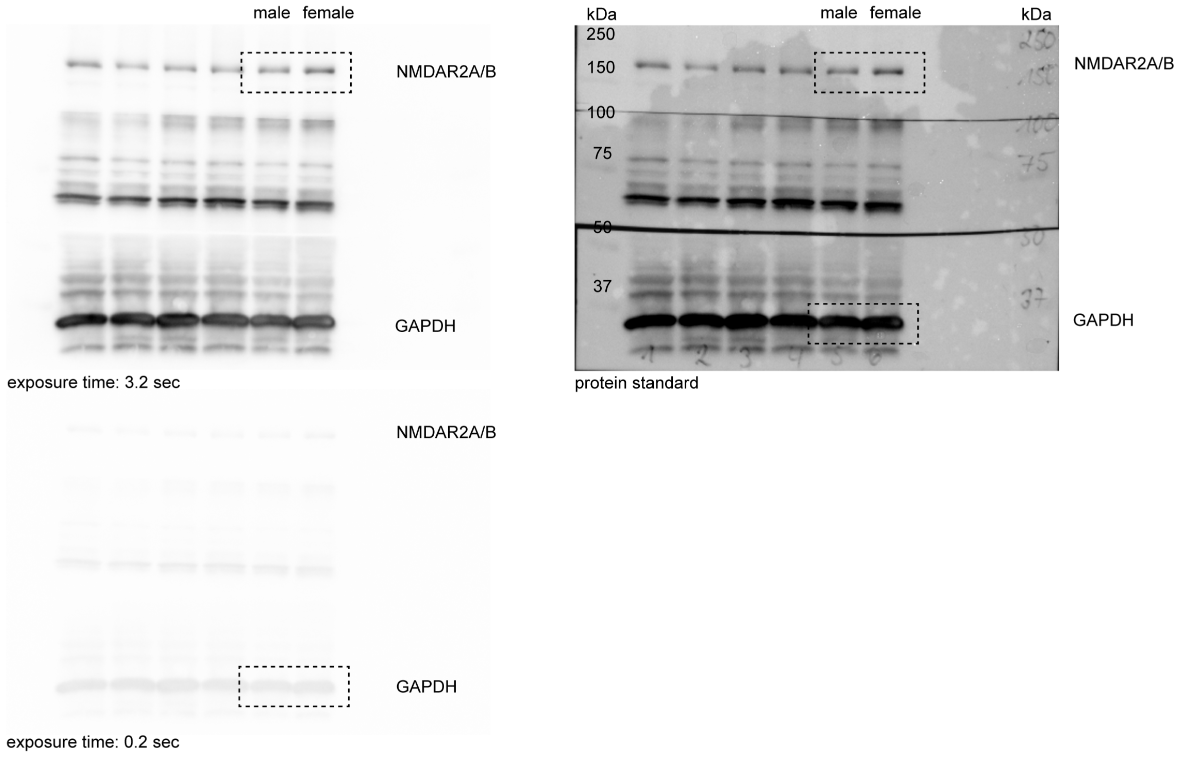

b

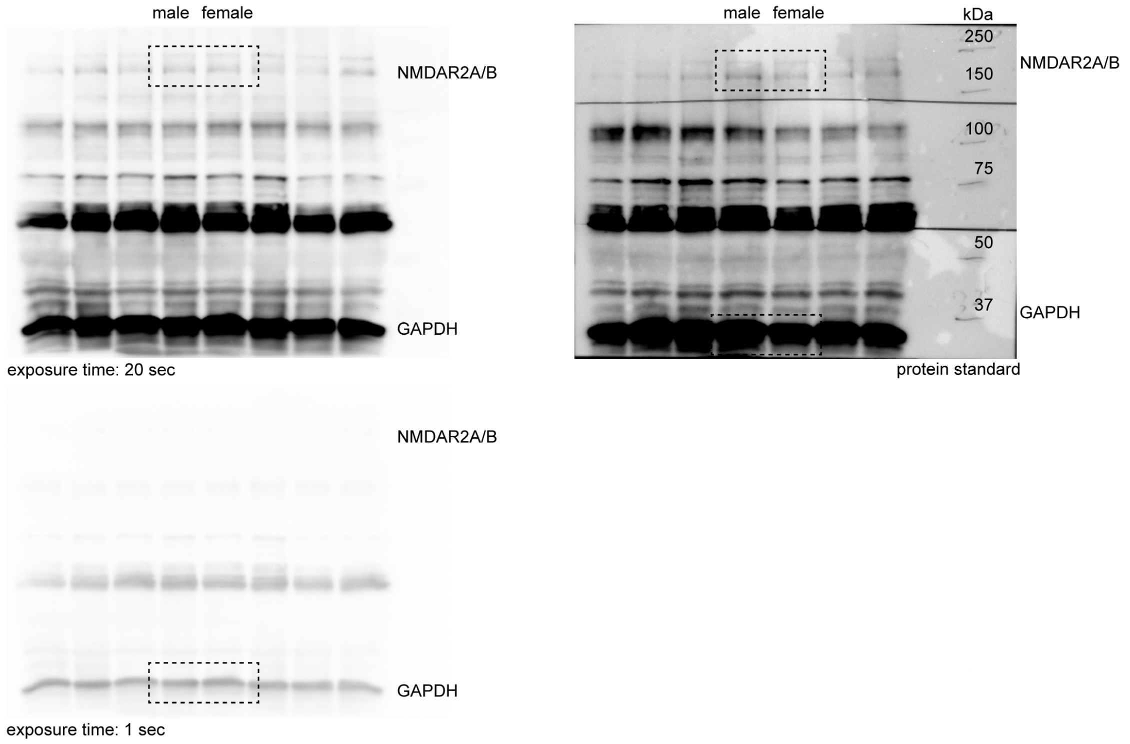

Supplemental data to Figure 4

c

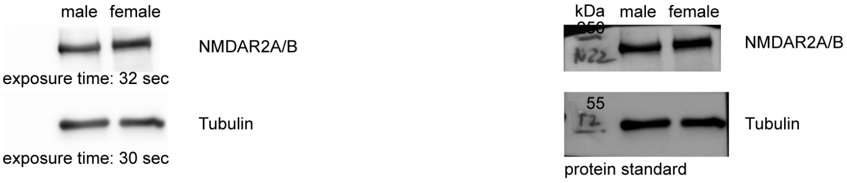

d

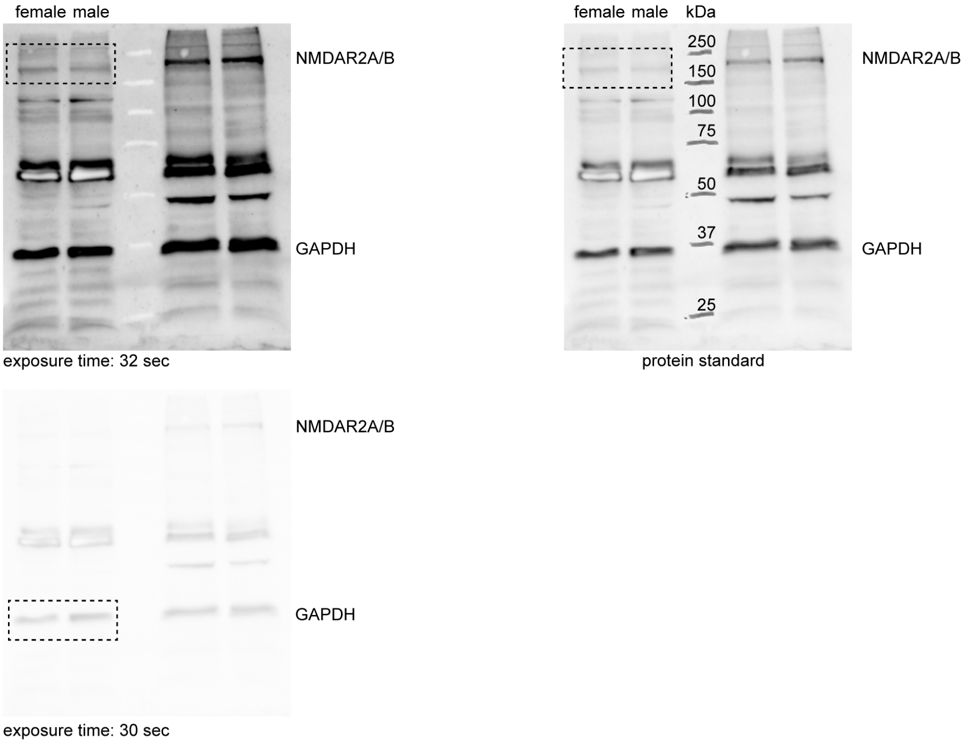

e

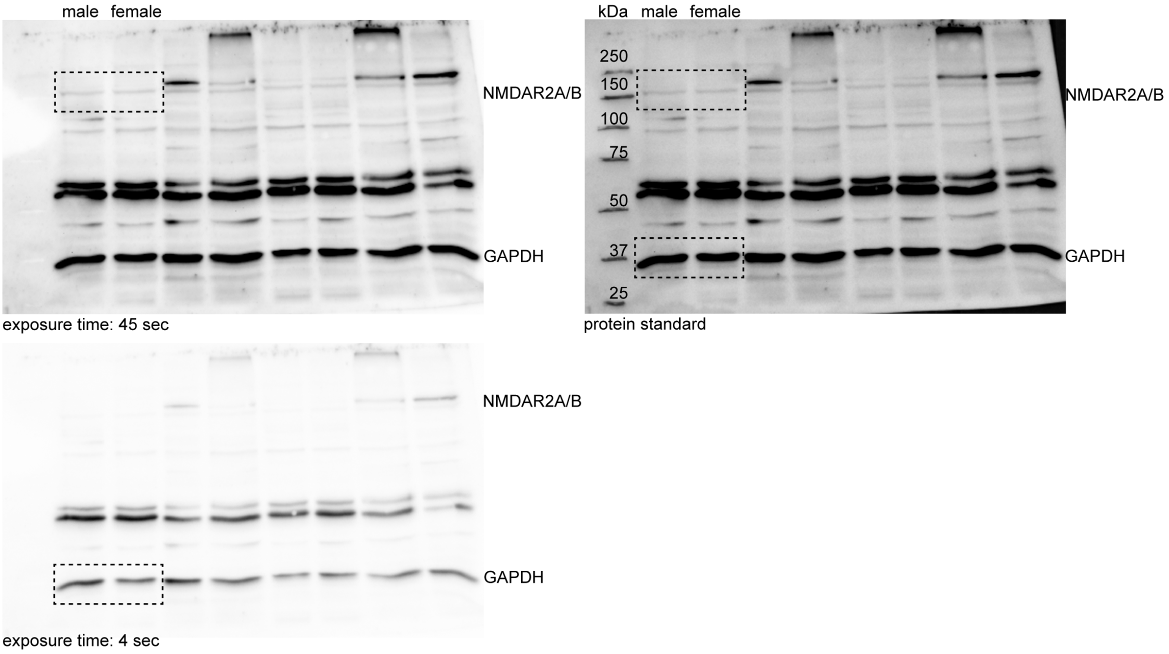

Supplement: Supplementary file 1 — Supplementary information [file 41598_2020_68371_MOESM1_ESM.pdf]
